# Energy Measures as Biomarkers of SARS-CoV-2 Variants and Receptors

**DOI:** 10.3390/bioengineering13010107

**Published:** 2026-01-16

**Authors:** Khawla Ghannoum Al Chawaf, Salim Lahmiri

**Affiliations:** Department of Supply Chain and Business Technology Management, John Molson School of Business, Concordia University, Montreal, QC H3H 0A1, Canada; khawla.ghannoumalchawaf@mail.concordia.ca

**Keywords:** SARS-CoV-2, COVID-19, statistical analysis, variant identification, human receptors, genetic sequences, multiple ANOVA test, Bartlett’s test

## Abstract

The COVID-19 outbreak has made it evident that the nature and behavior of SARS-CoV-2 requires constant research and surveillance, owing to the high mutation rates that lead to variants. This work focuses on the statistical analysis of energy measures as biomarkers of SARS-CoV-2. The main purpose of this study is to determine which energy measure can differentiate between SARS-CoV-2 variants, human cell receptors (GRP78 and ACE2), and their combinations. The dataset includes energy measures for different biological structures categorized by variants, receptors, and combinations, representing the sequence of variants and receptors. A multiple analysis of variance (ANOVA) test for equality of means and a Bartlett test for equality of variances are applied to energy measures. Results from multiple ANOVA show (a) the presence of significant differences in energy across variants, receptors, and combinations, (b) that average energy is significant only for receptors and combinations, but not for variants, and (c) the absence of significant differences observed for standard deviation across variants or combinations, but that there are significant differences across receptors. The results from the Bartlett tests show that (a) there is a presence of significant differences in the variances in energy across the variants and combinations, but no significant differences across receptors, (b) there is an absence of significant differences in variances across any group (variants, receptors, combinations), and (c) there is an absence of significant differences in variances for standard deviation of energy across variants, receptors, or combinations. In summary, it is concluded that energy and mean energy are the key biomarkers used to differentiate receptors and combinations. In addition, energy is the primary biomarker where variances differ across variants and combinations. These findings can help to implement tailored interventions, address the SARS-CoV-2 issue, and contribute considerably to the global fight against the pandemic.

## 1. Introduction

The scientific community faces a formidable foe in the relentless battle against the global COVID-19 pandemic: the ever-evolving severe acute respiratory syndrome coronavirus 2 (SARS-CoV-2). The virus’s unrelenting advance over the last three years has been fueled by its infectious nature and its capacity for constant mutation. These genetic differences have emerged as important determinants of the biological activities of the virus, regulating immune evasion and transmission dynamics, especially within the amino acid sequence of the surface spike (S) protein. As a result, the discovery and comprehension of these changes have become essential for developing focused treatment plans and implementing exact preventative and control measures around the globe.

In this regard, various studies have been conducted. For instance, Attiq et al. [1] used integrated machine learning (ML) templates as prediction tools to investigate the anti-SARS-CoV-2 significant protease potential of FDA-approved marine medicines. The results revealed that Holichondrin B’s consistent interaction with the SARS-CoV-2 major protease makes it a suitable lead drug for further investigation and possible clinical trials. The substance interacts with essential residues involved in protease activity, indicating that it may be used as a treatment, particularly for cancer patients’ COVID-19 symptoms. In [2], Qin et al. aimed to develop a rapid classification method for SARS-CoV-2 variant strains using a machine learning-based label-free surface-enhanced Raman scattering (SERS) strategy, since the timely discovery of these genetic variations and their interactions with human receptors is one of the main challenges in managing the COVID-19 pandemic effectively. In their study, SERS and logistic regression (LR) were the primary models applied. They concluded that the SERS-LR model can categorize variations in intricate biological materials. In this regard, they concluded that machine learning-based SERS technology can distinguish between different SARS-CoV-2 variants correctly and may be used for quick diagnosis and treatment selection. Torun et al. [3] proposed a meta-surface biosensor used to enable direct detection of SARS-CoV-2 in raw saliva. The three models used include computational screening of gold meta surfaces, ML classifiers for differentiating viral variants, and quantitative determination of viral concentration. Specifically, ML was used to detect SARS-CoV-2 from Raman spectra with high sensitivity and specificity while optimizing light–virus interaction, which is essential for molecular-level detection. Specifically, the goal was to use ML to distinguish between different viral variations, such as wild-type, alpha, and beta. The precision of the biosensor in identifying viruses and differentiating between variants is shown by validation using 36 positive and 33 negative clinical samples. With a sensitivity and specificity of 95.2%, the authors concluded that ML allows for the quantitative determination of viral concentration. In addition, they concluded that the research highlights the biosensor’s capacity for large-scale screening in raw saliva, providing a quick and precise preventative measure for controlling COVID-19.

Lee and Chen [4] addressed the application of deep learning (DL) to identify new drugs to treat COVID-19. They used gated recurrent units and other transformer-based models to explain large, complex molecular and clinical datasets. A significant component of their strategy concerned the multimodal analysis of protein sequences, three-dimensional structures, and gene expression patterns. The study’s integral conclusions involved identifying several new drugs with possible repurposing against SARS-CoV-2. Moreover, the authors identified the possibility of using DL to determine potential therapeutic agents quickly, which is especially valuable for responding to emerging virus variants. The models’ accuracy in predicting drug–target interactions and possible side effects gave them essential data to guide candidates for clinical trials. Titus et al. [5] discussed the various electrochemical biosensors’ architectures for identifying SARS-CoV-2 and examined the application of ML algorithms for increasing the sensitivities and accuracy of the detection systems. Such intelligent biosensors as artificial neural networks and support vector machines were employed for signal analysis of these biosensors. The authors also concluded that enhancing the biosensors with enhanced designs synchronized with the workings of ML algorithms significantly improved the speed and efficiency of testing for SARS-CoV-2. The sensitivity of some of the reviewed systems showed that viral particles could be detected at a femtomolar level. In addition, the authors pointed out the need for these integrated approaches to offer a fast diagnostic solution on the patient’s side where various SARS-CoV-2 variants can be identified. In [6], the authors aimed to create a new computational method for analyzing the movement of viral proteins and possible interactions with host cells. The cellular automata model was applied to explore the envelope protein under different situations to understand structural changes in the SARS-CoV-2 envelope protein, which could help investigate virus–host interactions further and design appropriate therapies. For instance, they used cellular automata enhanced machine learning (CAML) model to study biological strings. The main hypothesis was that large differences in the CAML model parameters of CoV-2 and CoV characterize the deviation in structure and function of envelope proteins in respect to the interaction of the virus with the host Golgi complex. The authors concluded that the difference in CAML is reflected in the contribution of the envelope protein towards the overall large difference in transmissibility of CoV-2 and CoV.

Rampogu et al. [7] studied marine drugs as therapeutic agents against SARS-CoV-2 by employing the essence dynamics and analysis of the free energy landscape. The authors planned to recognize prospective drug molecules of marine origin capable of interacting with the proteins of SARS-CoV-2. In this regard, they examined the interactions of marine compounds with viral proteins through molecular dynamics simulations and machine learning. They applied principal component analysis and free energy calculations of the complexes formed between the drug and the protein. Then, they identified several components from marine sources with good anti-SARS-CoV-2 inhibitory activity. The authors showed the importance of integrating computational approaches with machine learning solutions for the drug design against SARS-CoV-2 and its spawns. Vangipuram and Appusamy [8] proposed a machine learning-based COVID-19 diagnosis system. Using several machine learning algorithms, their study sought to develop a correct and swift diagnostic tool. The authors used decision trees, random forests, and support vector machine analyses on clinical and demographic datasets. Some of the highlights of their methods entailed the feature selection methods that would help to determine the most critical predictors of COVID-19 infection. This yielded a diagnostic model with impressive accuracy, sensitivity, and specificity. They concluded that machine learning can help create fast and practical diagnostic tests for COVID-19 that can be modified to identify other virus versions. Parvathy et al. [9] used machine learning models for the early detection of potential patients with long-term complications caused by COVID-19. The authors utilized logistic regression, random forest, and neural network models. In addition, features analysis was applied to patients’ clinical, laboratory, and demographic records. It was concluded that machine learning could predict the pathophysiology of SARS-CoV-2 infection, which would be helpful, especially when dealing with various virus strains. Gantini and Christian [10] used data mining techniques to analyze the 3D protein models of the SARS-CoV-2 virus. Their study explored primary structural properties and searched for common overall structural themes in viral proteins that could be utilized in designing effective vaccines and drugs. For instance, they employed clustering and association rules on the protein’s sequence and structure data to determine regions in different strains of the virus that may also hold the epitopes for the targets that can be vaccinated. In addition, they discovered several promising protein regions for subsequent analysis. They concluded that clustering and association rules can be applied at the molecular level concerning SARS-CoV-2, which might help discover effective treatments and vaccines for various stemming from various mutations.

The ongoing evolution of SARS-CoV-2 has led to the emergence of multiple variants, each exhibiting distinct transmissibility and pathogenicity profiles. Developing successful treatment plans requires an understanding of the molecular interactions between these variations and host cell receptors. The angiotensin-converting enzyme 2 (ACE2) is the main receptor that allows SARS-CoV-2 to enter host cells, but new research has shown that the glucose-regulated protein 78 (GRP78) is another receptor that may affect viral infectivity [11]. Understanding the mechanisms behind viral entrance and infection can be gained by examining the binding affinities and interaction energies between SARS-CoV-2 variants and these receptors. Prior studies have evaluated the binding free energies of several SARS-CoV-2 variants with ACE2 using computational techniques, identifying variations in binding affinities that could be associated with higher transmissibility [12]. Similarly, studies have explored the interaction between the receptor-binding domain (RBD) of the virus and GRP78, suggesting a potential role for GRP78 in mediating viral entry [13].

In this study, we seek to identify which energy measures can effectively differentiate between SARS-CoV-2 variants, the receptors ACE2 and GRP78, and their combinations. By applying both multiple ANOVA to test for equality of means and Bartlett’s test to assess the equality of variances, we seek to elucidate the statistical significance of observed differences in interaction energies. This comprehensive analysis will enhance our understanding of the molecular interactions governing SARS-CoV-2 infectivity and may inform the development of targeted therapeutic interventions. For instance, we need to identify which energy measures can effectively differentiate between SARS-CoV-2 variants, the receptors ACE2 and GRP78, and their combinations. Clarifying the molecular interactions that control viral entrance and infectivity requires an understanding of these differences. In this regard, we determine the statistical significance of observed variations in energy measures across SARS-CoV-2 variants, human cell receptors (GRP78 and ACE2), and their combinations. Specifically, we use Bartlett’s test to assess the equality of variances and several ANOVA tests to compare the means of interaction energies.

While recent works used statistical machine learning to detect SARS-CoV-2 [1,2,3,4,5,6,7,8,9,10] and conducted statistical tests to understand the mechanisms behind viral entrance and infection [11,12,13], our study contributes to the literature by conducting rigorous statistical analyses to deeply understand the molecular mechanisms underlying SARS-CoV-2 infectivity and potentially inform the design of targeted therapeutic interventions. Indeed, our statistical methodology seeks to improve the categorization system’s precision and flexibility. In addition, the study tackles the challenges associated with classifying variations within the SARS family, going beyond general classifications to thoroughly examine individual variants. A more profound knowledge of the virus’s infection processes is made possible by identifying and tagging specific human receptors, such as ACE2 and GRP78, and their unique families [14]. This allows for more focused therapies. These efforts resulted in the creation of an extensive eight-class system that captures the complex interactions between variations and human receptors. This study stands out because of its ambitious classification strategy, which has the potential to make significant progress in the area of SARS-CoV-2 variant identification. In sum, the work provides a more comprehensive knowledge of the virus and its interaction with human receptors, with implications for medicinal approaches and public health initiatives.

The rest of the paper is organized as follows. Section 2 presents the methodology. Section 3 presents data and provides statistical results. Finally, the discussion and conclusion are provided in Section 4.

## 2. Methods

The objective of this study is to determine which energy measure can be used as a biomarker to effectively differentiate between variants, receptors, and their combinations. We use statistical hypothesis testing techniques including multiple analysis of variance (ANOVA) tests [15,16] and Bartlett’s tests for equal variances [17]. These tests assess differences in energy measures across various categorical groups. Energy measurements for various biological structures are included in the collection, which is arranged according to receptors, variations, and their combinations.

**Figure 1:**
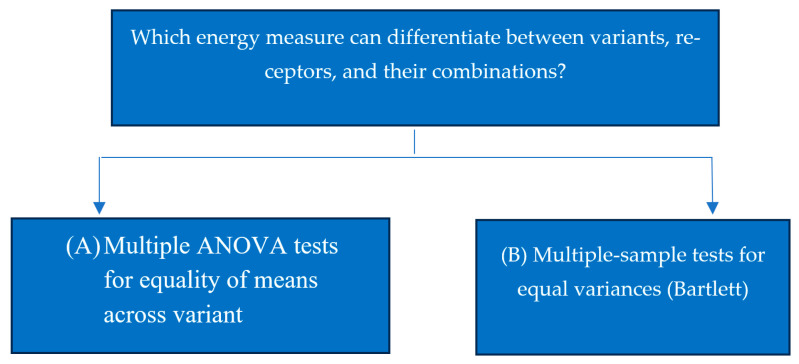
Flowchart of statistical analyses.

**Figure 2:**
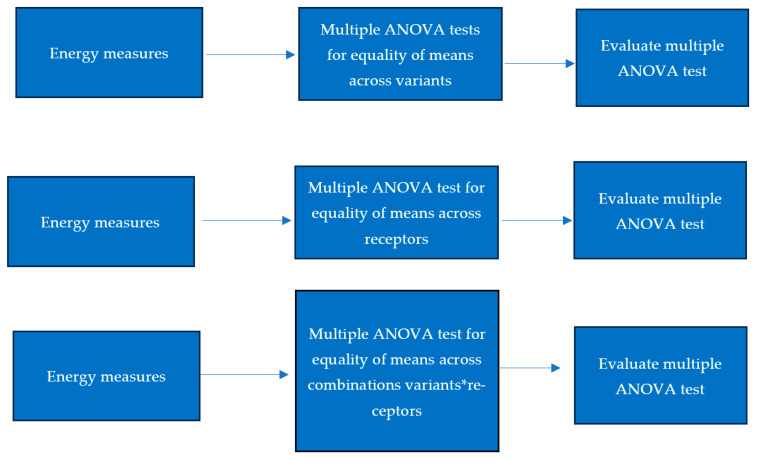
Multiple ANOVA tests for equality of means across variants.

To examine the variance and mean differences across categorical groups we use two main statistical tests: Bartlett’s test and multiple ANOVA. First, we conducted multiple ANOVA tests as shown in Figure 1. The ANOVA test shown in Figure 2 determines whether there are significant differences in the mean energy measures between the category groups. ANOVA is used in three situations:

1- ANOVA across variants: Examines if different variants show significant differences in mean energy measures.
2- ANOVA across receptors: tests for significant mean differences among receptors.
3- ANOVA across combinations of variants and receptors: Evaluates mean energy measure differences for the interaction between variants and receptors.

**Figure 3:**
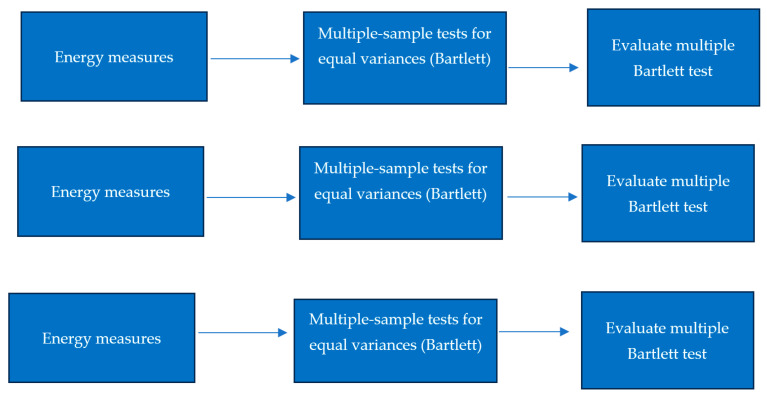
Multiple-sample tests for equal variances (Bartlett).

Then, we conducted Bartlett’s test for equal variances across the same categorical groups as shown in Figure 3:

1- Bartlett’s test across variants: assesses if variations in energy measures vary between variants.
2- Bartlett’s test across receptors: evaluates variance differences among receptors.
3- Bartlett’s test across combinations of variants and receptors: assesses variance homogeneity for interactions between variants and receptors.

## 3. Data and Results

The dataset comprised the RNA sequences of SARS-CoV-2 variants of concern, such as the Alpha, Beta, Gamma, Delta, Omicron, and others, and the human receptor sequences of ACE2 and GRP78. These sequences were retrieved from authentic genomic databases such as GISAID [18] or GenBank [19]. In the experiments, the RNA sequence for each SARS-CoV-2 variant for ACE2, GRP78, and wild-type RBD was downloaded and converted to a protein sequence. The target proteins in our experiment are the SARS-CoV-2 variants (Omicron, Delta, Gamma, Eta, Zeta, Iota, Beta, and Alpha), RBD, ACE2, and Grp78. Details on the sequencing follow.

The RBDs of the original SARS-CoV-2, the GRP78 region, and the common ACE2 allele were sequenced first as wild-types (WTs). However, a specific region for each was used in this experiment. The sequence from 334 to 530 in the wild-type RBD region was chosen based on its mutations within the ACE2 binding site that have appeared in many lineages/clades of SARS-CoV-2. For example, the N501Y mutation has appeared in Beta (B.1.351; 20 H/501Y.V2), Gamma (P.1; 20 J/501Y.V3), Alpha (B.1.1.7; 20I/501Y.V1), and Omicron (B.1.1.529; 21M/501Y). Therefore, the mutations of SARS-CoV-2 were found in this specific region of the RBD wild-type domain. The ACE2 region in this experiment is the region where it interfaces with SARS-CoV-2 RBD, in which 16 residues of SARS-CoV-2 RBD were shown to be in contact with 20 residues of ACE2 [20]. However, for Grp78 (also known as HSPA5), the region where Grp78 and SARS-CoV-2 RBD interact was sequenced and expressed.

SARS-CoV-2 variants were sequenced based on their mutations in the RBD. The Alpha variant has one mutation located on N501Y, and Zeta/Iota/Eta also have one on E484K; thus, they have the same sequence. Delta has two mutations located at L452R and T478K. Beta has three mutations located on K417N, E484K, and N501Y. Gamma has three mutations on K417T, E484K, and N501Y. However, Omicron has fifteen mutations located at G339D, S371L, S373P, S375F, K417N, N440K, G446S, S477N, T478K, E484A, Q493R, G496S, Q498R, N501Y, and Y505H.

Homology modeling is then used to generate a 3D protein model from the target sequence and evolutionary-related protein structures. Practical information will be gathered and used as a template. The first step in the SWISS-MODEL was the input of data: an amino acid sequence in FASTA text was input in the SWISS-MODEL of the target protein. UniProtKB was used to help build the FASTA text [21]. The target proteins in our experiment are the SARS-CoV-2 variants (Omicron, Delta, Gamma, Eta, Zeta, Iota, Beta, and Alpha), RBD, ACE2, and Grp78. The second step was to search the template using SWISS-MODEL. Two database search methods were used to perform this task: HHblits [22] and Blast [23]. In the case of remote homology, HHblits adds sensitivity; however, the BLAST method can find closely related templates, which provides high and fast accuracy. Therefore, the input data are used in this step to search for protein structures that are evolutionarily related to the input against the template library of the SWISS-MODEL (SMTL) [24].

The third step is template selection. After the templates are completed and estimated by the Quaternary Structure Quality Estimate (QSQE) [25] and Global Model Quality Estimate (GMQE) [26], and according to the expected quality of the resulting models, the templates are ranked [24]. For example, different models were built in the RBD, and many templates were selected automatically. So, to choose the best template, the SWISS-MODEL provides many alternative template options. Each template has a descriptive set of features that allows the user to select the best fit with the target protein. So, template 6vw.1.1. B was used in the RBD because it is a SARS-CoV-2 chimeric receptor-binding domain complexed with its human receptor ACE2. This template was selected because of its defining features and good interactive graphical views.

A 3D protein model is generated after selecting the template as defined by the alignment of the target template to conserved atom coordinates. A full-atom protein model and loop modeling generates the residue coordinates from the constructed amino acid non-conserved side chains. The SWISS-MODEL relies on the ProMod3 modeling engine and the OpenStructure computational structural biology framework to perform this step [27]. After building models for the target proteins using the SWISS-MODEL, PDB files were downloaded and checked on the PyMOL 2.6 System.

After obtaining the PDF files, they were submitted to docking software known as SwarmDock. SwarmDock is a memetic docking algorithm in which the conformational, orientational, and translational degrees of freedom were developed using normal modes to perform flexible docking, which are then simultaneously optimized with a Solis and Wets local search algorithm using the Particle Swam Optimization (PSO) metaheuristic [28]. SwarmDock was used instead of other algorithms due to its simplistic energy function, in which, upon binding, it undergoes significant conformational changes; thus, it can dock flexible structures successfully.

Compared to other docking methods, SwarmDock is different in that it filters the many putative structures using FFT correlations or combines the single independent trajectories’ results using search space [28]. As an emergent proportion of the system, the exploitation of narrow regions containing lower energy structures is switched between them and the exploration of diffuse areas in search space, which depend on the nature of the energy of the landscape. Surrounding the binding site, a correlated energy landscape is used by SwarmDock as swarm members find low-energy positions that act as attractors for some swarm members. Furthermore, the equation describing the velocities of the swarm members has a spatially varying repulsion term that prevents the contraction of a dispersed swarm from occurring. When a swarm concentrates its efforts on one specific location with many low-energy structures, such as the actual biological interface, it has less impact on its contraction [28].

In the docking technique, a standard energy function is incorporated. The approach employs van der Waals and Coulombic terms. These terms are found between the i and j associative atoms within the receptor and ligand, respectively [28]. Also, a switching function between 7 and 9 (Ron = 7 and roff = 9) is used to eliminate any interruptions in the standard relation and prevent long-distance mathematical associations with an insignificant contribution to the interaction energy [28].

A standard energy function is computed based on van der Waals and Coulombic terms [28]. For instance, let *i* and *j* be associative atoms within the receptor and ligand, respectively [28]. Then, the energy function in kilo Joule per mole is given by the following [28]:

(1) $$E_{\mathrm{int}}=\;\sum_{i}^{atoms}\times \sum_{j}^{atoms}\times E_{i,j}$$

**Figure 4:**
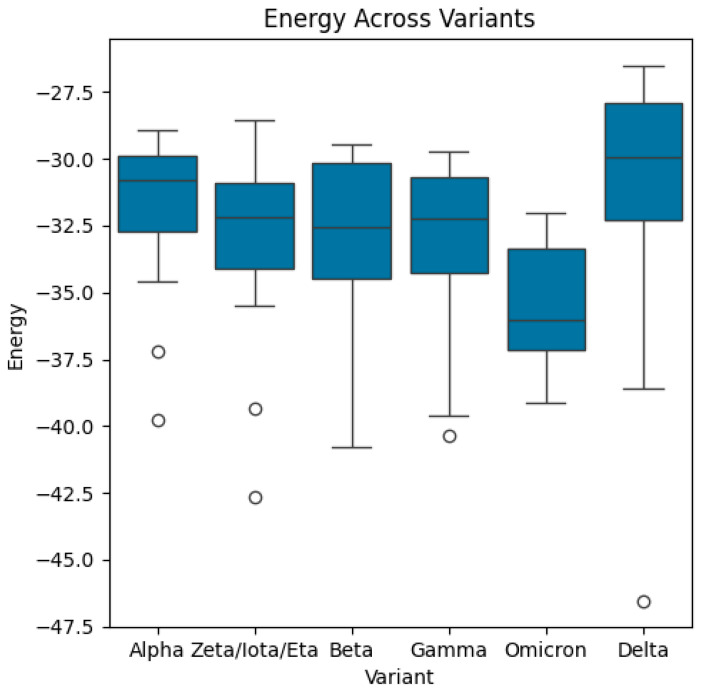
Boxplots of energy across variants. The median energy of the Delta variant is the highest, whilst the median energy of Omicron is the lowest. The highest and lowest values of energy are associated with the Delta variant. The highest and lowest quartiles are, respectively, associated with the Delta variant and Omicron variant.

**Figure 5:**
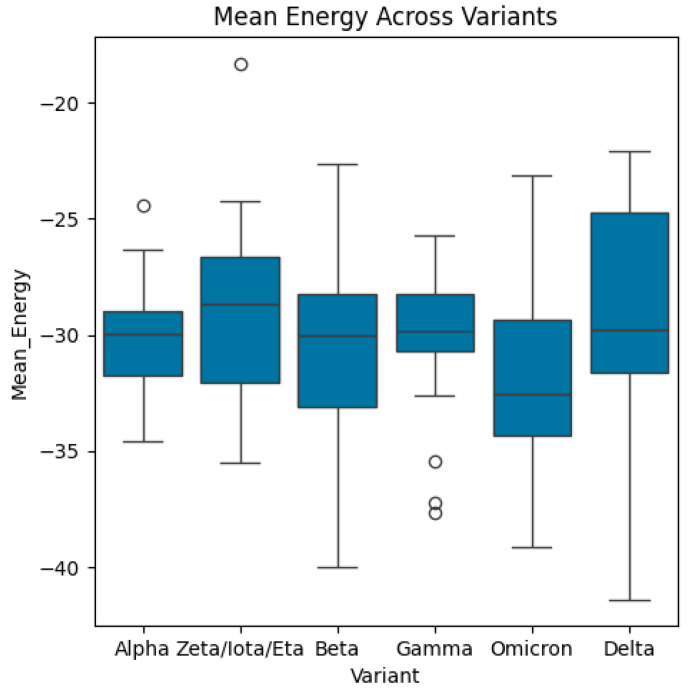
Boxplots of mean energy across variants. The median of mean energy of the Delta variant is the highest, whilst the median energy of Omicron is the lowest. The highest and lowest values of mean energy are associated with the Delta variant. The highest and lowest quartiles are, respectively, associated with Delta variant and Omicron variant. The Gamma variant shows three outliers.

**Figure 6:**
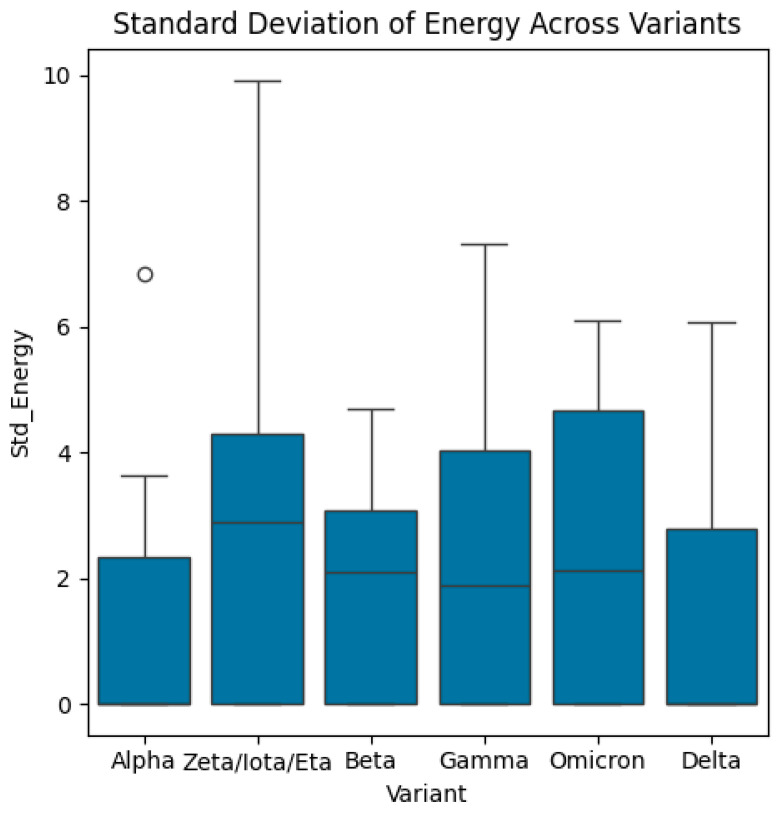
Boxplots of standard deviation of energy across variants. The median standard deviation of energy in Zeta/Iota/Eta variant is the highest. The median standard deviation of energy of the Gamma variant is the lowest. The highest value of standard deviation of energy is associated with Zeta/Iota/Eta variant. The Alpha variant shows only one outlier.

**Figure 7:**
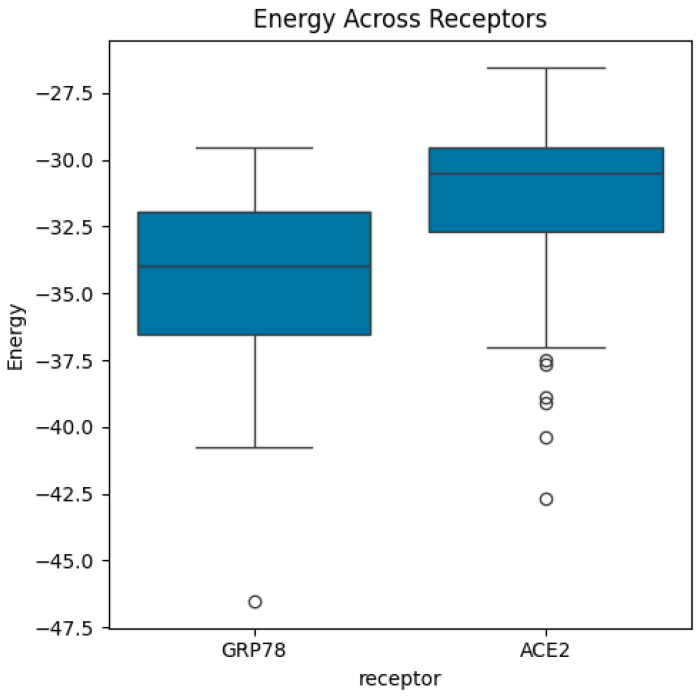
Boxplots of energy across receptors. The median of energy in the ACE2 receptor is larger than the median of energy in GRP78 receptor. The highest and lowest energy values are associated, respectively, with ACE2 receptor and GRP78 receptor. ACE2 receptor shows several outliers whilst GRP78 shows only one outlier corresponding to the lowest energy value.

**Figure 8:**
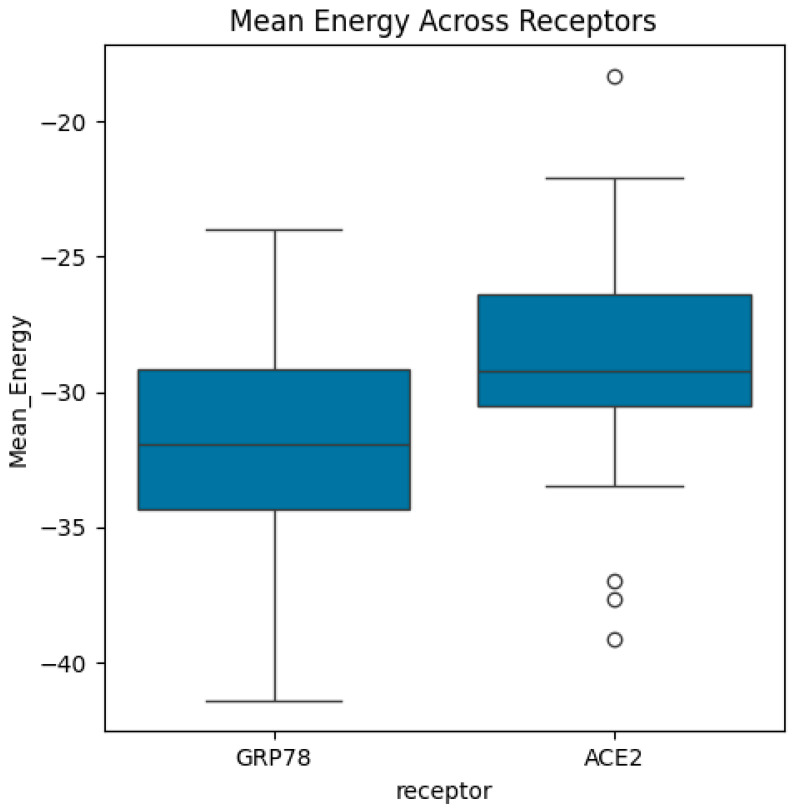
Boxplots of mean energy across receptors. The median of mean energy in ACE2 receptor is larger than the median of mean energy in GRP78 receptor. The highest and lowest mean energy values are associated with ACE2 receptor and GRP78 receptor, respectively. ACE2 receptor shows several outliers.

**Figure 9:**
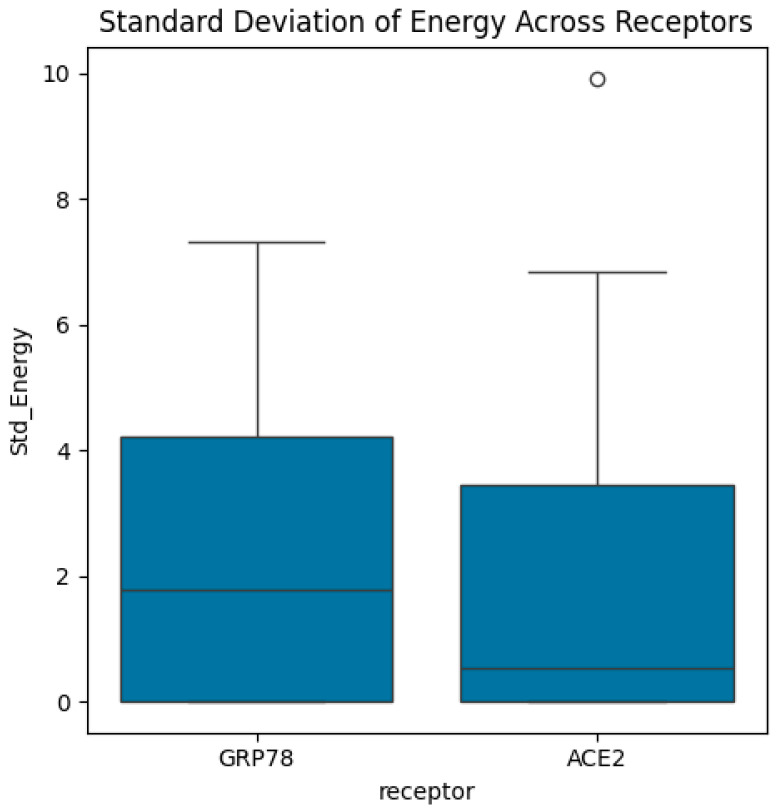
Boxplots of standard deviation of energy across receptors. The median standard deviation of energy in the GRP78 receptor is higher than the median standard deviation of energy in the ACE2 receptor. The highest standard deviation in measured energy is associated with the ACE2 receptor, which corresponds to its unique outlier.

Then, the dataset includes energy measures for different biological structures categorized by variants, receptors, and combinations, representing the sequence of variants and receptors. In this study, we computed the mean and standard deviations of energy function (see Equation (1)) for each variant and receptor. Also, we calculated the mean/average of mean energy across all variants and mean/average of mean energy across all receptors. The distributions of energy, mean energy, and standard deviation of energy are exhibited in Figure 4, Figure 5, Figure 6, Figure 7, Figure 8 and Figure 9.

The results from all statistical tests are provided in Table 1 and Table 2 where all tests are applied at 5% significance level with test-associated probability values (*p*-value). Based on the results from the multiple ANOVA tests when applied to variants (Table 1), the main findings are as follows. The ANOVA test shows that the mean energy differs significantly across the variants (*p*-value = 3.0636 × 10^−3^). In addition, it shows no significant difference in the mean/average of mean energy across the variants (*p*-value = 1.2023 × 10^−1^). Furthermore, according to the ANOVA test, there is no significant difference in the energy across the variants (*p*-value = 1.9736 × 10^−1^). In addition, the results from multiple ANOVA tests when applied to receptors (Table 1) show that the mean energy differs significantly across the receptors (*p*-value = 5.5317 × 10^−6^), with a significant difference in the mean/average of mean energy across the receptors (*p*-value = 2.3903 × 10^−5^), and no significant difference in the standard deviation of energy across the receptors (*p*-value = 4.6348 × 10^−1^). Finally, the results from multiple ANOVA tests when applied to combinations of variants and receptors (Table 1) show that the energy values differ significantly across the combinations of variants and receptors (*p*-value = 2.1419 × 10^−7^), the mean energy differs significantly across the combinations of variants and receptors (*p*-value = 9.1950 × 10^−5^), and there is no significant difference in the standard deviation of energy across the combinations of variants and receptors (*p*-value = 3.4097 × 10^−1^).

In short, the findings from the ANOVA tests (Table 1) can be summarized as follows. First, we found significant differences in energy across variants, receptors, and combinations. Second, we found that the average energy is significant only for receptors and combinations, but not for variants. Third, there are no significant differences observed for standard deviation across variants or combinations, but these were significant across receptors. As a result, energy and mean energy are the key biomarkers used to differentiate receptors and combinations, while the standard deviation of energy does not show significant differences in most cases.

Based on the results from the multiple Bartlett tests when applied to variants (Table 2), the main findings are as follows. The variances in energy are significantly different across the variants (*p*-value = 4.2953 × 10^−2^), the variances in the mean energy are not significantly different across the variants (*p*-value = 7.0220 × 10^−2^), and the variances in energy are equal across the two receptors (*p*-value = 5.5891 × 10^−1^). Based on the results from the multiple Bartlett tests when applied to receptors (Table 2), it is found that the variances in energy are equal across the two receptors (*p*-value = 9.4828 × 10^−1^), the variances in mean energy are equal across the two receptors (*p*-value = 8.2845 × 10^−1^), and there is no significant difference in the standard deviations of energy across the two receptors (*p*-value = 8.4075 × 10^−1^). Finally, when multiple Bartlett tests are applied to combinations, it is found that the variances in energy differ significantly across the combinations of variants and receptors (*p*-value = 3.2958 × 10^−5^), the variances in mean energy are not significantly different across the combinations (*p*-value = 2.7196 × 10^−1^), and there is no significant difference in the standard deviations of energy across the combinations of variants and receptors (*p*-value = 6.7844 × 10^−1^).

In short, the results from multiple Bartlett tests can be summarized as follows. For energy biomarkers, there are significant differences in the variances in energy across the variants and combinations, but no significant differences across receptors. For mean energy biomarkers, there are no significant differences in variances across any group (variants, receptors, and combinations). For the standard deviation of energy as a biomarker, there are no significant differences in the variances for the standard deviation of energy across variants, receptors, or combinations. In sum, energy biomarker is the primary factor where variances differ across variants and combinations, while mean energy and standard deviation of energy show no significant differences.

## 4. Discussion and Conclusions

There is abundant literature on the statistical analysis and prediction of SARS-CoV-2, including, for instance, the development of deep learning models for precise segmentation of COVID-19 lesions in chest computed tomography (CT) scans [29,30]; identification of at-risk patients diagnosed with COVID-19 or related infections [31]; evaluation of short-term clinical outcomes in COVID-19 pneumonia patients [32]; direct quantification of respiration by performing segmentation based on respiratory images [33]; analysis of key health outputs, such as discharge conditions, mortality, and COVID-19 cases, with the goal of improving responses to future crises [34]; understanding the characteristics of microcirculation that are mainly affected by COVID-19 infection [35]; predicting the development of the Omicron epidemic [36]; cough-based diagnosis for respiratory diseases on COVID-19 patients [37]; analysis of ultra-low-dose X-ray images to detect COVID-19 [38]; and using multi-ligand virtual screening to identify small molecule inhibitors for their efficacy against SARS-CoV-2 virus [39], to name few.

This work focuses on the statistical analysis of energy measures as biomarkers of SARS-CoV-2. The main purpose of this study is to determine which energy measure can differentiate between SARS-CoV-2 variants, human cell receptors (GRP78 and ACE2), and their combinations. The main goals of our study are as follows. First, we analyze and compare the interaction energies between different SARS-CoV-2 variants and the ACE2 and GRP78 receptors. Second, we investigate whether combinations of these variants and receptors exhibit distinct energy profiles that could influence viral binding and entry. Third, we assess the homogeneity of variances across groups using Bartlett’s test, ensuring the validity of the ANOVA assumptions. Fourth, we identify which energy measure (e.g., binding free energy, mean interaction energy, or standard deviation) provides the most reliable differentiation between variants, receptors, and their combinations.

The results from both the ANOVA and Bartlett tests provide valuable insights into the differences in energy measures across SARS-CoV-2 variants, receptors, and their combinations. The findings suggest that energy and mean energy are the key differentiators in receptor and combination interactions, whereas the standard deviation of energy does not exhibit significant variability in most cases. These results have important implications for understanding how viral variants interact with host receptors and may inform the development of therapeutic strategies targeting these interactions.

The ANOVA results revealed significant differences in energy across variants, receptors, and their combinations, which led to the rejection of the null hypotheses for these groups. This indicates that the interaction energies between SARS-CoV-2 variants and receptors differ significantly, supporting the idea that these energy measures can be used to distinguish between different viral strains and their potential to bind to host receptors. The significant differences in mean energy between receptors and combinations further highlight the relevance of energy as a distinguishing feature in viral entry. However, the lack of significant differences in mean energy for variants suggests that mean energy may not be a suitable differentiator for variants alone, emphasizing the importance of considering receptor interactions and their combinations in understanding viral binding.

The standard deviation of energy, however, did not show significant differences across variants or combinations in the ANOVA analysis. This suggests that the variability in energy interactions is relatively consistent across these groups, implying that standard deviation is not a useful measure for distinguishing between them. Interestingly, the Bartlett test showed significant differences in the variances in energy across variants and combinations but not across receptors. This finding indicates that while the energy values themselves differ significantly, the variance in these values is more pronounced when comparing different variants and their combinations with receptors. The lack of significant variance differences across receptors suggests that the receptor’s role in the interaction does not vary as much as the variants themselves, potentially reflecting the more consistent binding properties of ACE2 and GRP78 compared to the dynamic nature of SARS-CoV-2 variants.

Moreover, the Bartlett test showed no significant differences in the variances in mean energy or standard deviation of energy across the groups, further emphasizing that these measures do not exhibit substantial variation across variants, receptors, or combinations. The absence of significant variance differences in mean energy and standard deviation suggests that while energy is a clear differentiator, the variability within these measures is minimal, reinforcing the robustness of energy as a key factor in distinguishing viral-receptor interactions.

It is worth noting that Bartlett’s test indicates heterogeneity of variances for energy across all variants and for energy across all combinations. Since the data size is perfectly balanced across variants and receptors, the effect of heterogeneity of variances on standard ANOVA tests would be very limited on these sets. Also, it is worth mentioning that all ANOVA tests are performed under the normality assumption. Because of limited samples sizes, the results could be considered as explanatory. In addition, the calculated average energy by variant/receptor presented in Table 3 shows that Omicron/GRP78 achieved the highest energy in absolute value (−33.4403) followed by Beta/GRP78 (−33.0438) and Delta/GRP78 (−32.6514), whilst Delta/ACE2 obtained the lowest average energy in absolute value (−26.1153) followed by Zeta/ACE2 (−27.9563) and Beta/ACE2 (−28.2156). Finally, Gamma/GRP78 and Gamma/ACE2 exhibit moderate average energy. For instance, they yielded to −30.3382 and −30.1941, respectively.

In summary, the findings indicate that energy is the most significant factor in differentiating between SARS-CoV-2 variants, receptors, and their combinations. While mean energy shows some discriminative power, especially in receptor and combination groups, the standard deviation of energy does not offer substantial differentiation. These results underline the importance of energy as a measure for assessing viral binding and entry and suggest that energy-based analysis could be a promising approach for understanding SARS-CoV-2 infectivity. However, further research is needed to explore the biological relevance of these energy measures and to validate these findings experimentally.

Overall, this study not only improves our knowledge of the interactions between SARS and CoV-2 variants but also offers useful information that may influence future treatment and prevention plans for both present and future coronavirus threats. Indeed, our work offers several significant contributions to the field of virology and molecular biology. First, it offers a better knowledge of the molecular mechanisms controlling viral binding and entrance by examining the energy interactions between SARS-CoV-2 variants and the host receptors ACE2 and GRP78. This insight is crucial for explaining differences in infectivity and transmissibility among variants. Second, utilizing multiple ANOVA tests to assess differences in means and Bartlett’s test to check the equality of variances ensures a rigorous statistical comparison of interaction energies. This scientific technique aids in determining the most discriminative energy metrics and improves the findings’ dependability. Third, targeted antiviral treatments can be developed with knowledge of which energy measurements can distinguish between variations and receptor contacts. New therapeutic approaches targeted at preventing viral entrance may be made possible by understanding the binding kinetics of GRP78, a developing alternative receptor. Fourth, the results of the study on differential binding energies can help anticipate if new SARS-CoV-2 variants are transmissible and harmful. Through the identification of variations with higher binding affinities that may provide increased transmission risks, this could be useful for vaccine creation and public health efforts.

Overall, our statistical findings suggest that the physical and chemical binding energy information adds an extra layer of biological relevance, even though most of the previous research focuses on classifying variations based only on sequence changes [40]. Indeed, incorporating these energy metrics can potentially enhance differentiation and provide a more stable and biologically meaningful basis for assessing variant behavior. In addition, we found that there are significant differences in energy interaction between various viral strains. According to Nguyen et al. [41], this discovery advances our understanding of how structural modifications to the viral spike protein affect binding energetics and, consequently, infectivity. Furthermore, we found that mean energy distinguishes between the ACE2 and GRP78 receptors, which offers a thermodynamic explanation for differences in receptor vulnerability. Previous studies have focused on the structural compatibility of the receptors [42], but this work extends that knowledge by statistically validating the energetic differences in their interactions. Moreover, we found a significant combined impact of viral evolution and receptor diversity on binding behavior by enabling the identification of energy parameters that are sensitive to both the host receptor and the viral variation. Such insights have been largely absent from previous computational studies, which typically analyze variant–receptor interactions in isolation [43].

The implications of this study extend beyond theoretical knowledge, impacting both clinical and public health domains. First, the discovery lays the groundwork for developing small compounds or antibodies that can interfere with variant–receptor contacts and possibly stop viral entrance by identifying energy metrics that significantly differentiate these interactions. Second, proactive public health interventions may be made possible by the identification of energy profiles linked to highly transmissible variations, which could help develop early warning systems for new strains. Third, to improve cross-variant protection, vaccine developers can optimize immunogens that target conserved areas involved in receptor binding by using insights into how various variants interact with ACE2 and GRP78. Fourth, to better understand how mutations impact virus–host interactions and support the study of viral evolution, the analytical framework used in this work can be extended to different viral systems.

This study lays the groundwork for understanding the interaction energies between SARS-CoV-2 variants and the receptors ACE2 and GRP78, but there are several avenues for future research that could deepen and expand these insights. One important direction is the validation of computational findings through experimental methods. Laboratory-based assays, such as surface plasmon resonance or isothermal titration calorimetry, could provide direct measurements of binding affinities, confirming the interaction energies predicted in this study. Additionally, using cell-based systems to observe viral entry and infectivity would help link the computational data to real-world biological outcomes. This combined approach would strengthen the validity of the results and bridge the gap between theoretical predictions and experimental reality. Expanding the scope of the study to include other receptors and co-receptors involved in SARS-CoV-2 entry is another valuable direction. While ACE2 and GRP78 are significant, emerging research suggests that other proteins, such as Neuropilin-1 and CD147, might also facilitate viral entry. Investigating these additional receptors could provide a more comprehensive understanding of the virus–host interaction network and identify alternative therapeutic targets. Furthermore, as new SARS-CoV-2 variants continue to emerge, it is crucial to apply this study’s framework to analyze these new strains. By comparing their interaction energies with those of previously studied variants, researchers can track evolutionary changes in binding characteristics that may influence transmissibility and pathogenicity.

Another promising direction is to explore the influence of environmental and biological factors on interaction between energies. For example, investigating how changes in pH, temperature, or glycosylation patterns affect the binding affinities could provide more realistic models of viral entry. Additionally, studying these interactions in the context of membrane microenvironments or in the presence of other host proteins would better reflect the complexity of cellular systems. To enhance the statistical robustness of future analyses, more advanced models, such as mixed-effects models or machine learning algorithms, could be employed to account for non-linear interactions and potential confounding factors.

Finally, extending this research to investigate therapeutic interventions is a logical next step. By identifying energy measures that significantly differentiate between variants and receptors, future studies could design small molecules, peptides, or antibodies to disrupt these interactions. This could pave the way for novel antiviral therapies that specifically target variant-specific binding mechanisms. In conclusion, while this study provides a strong foundation, pursuing these future research directions will lead to a more comprehensive understanding of SARS-CoV-2 variant interactions and potentially inform the development of effective therapeutic and preventive strategies.

## Figures and Tables

**Table 1:** Multiple ANOVA test results.

Null Hypotheses (H0) and Variables	*F*-Statistic	*p*-Value
**Tests applied to variants**		
H0: Energy is equal across all variants	3.8249	3.0636 × 10^−3^
H0: Mean of energy is equal across all variants	1.7909	1.2023 × 10^−1^
H0: Standard deviation of energy is equal across all variants	1.4937	1.9736 × 10^−1^
**Tests applied to receptors**		
H0: Energy is equal across both receptors	22.6625	5.5317 × 10^−6^
H0: Mean of energy is equal across both receptors	19.3578	2.3903 × 10^−5^
H0: Standard deviation of energy is equal across both receptors	0.5410	4.6348 × 10^−1^
**Tests applied to combinations**		
H0: Energy is equal across all combinations	5.8637	2.1419 × 10^−7^
H0: Mean of energy is equal across all combinations	3.9013	9.1950 × 10^−5^
H0: Standard deviation of energy is equal across all combinations	1.1360	3.4097 × 10^−1^

The total number of samples is 120. The number of samples in each variant and in each receptor is 10. All statistical tests are performed at 5% significance level.

**Table 2:** Multiple Bartlett test results.

Null Hypotheses (H0) and Variables	*F*-Statistic	*p*-Value
**Tests applied to variants**		
H0: Energy is equal across all variants	11.4619	4.2953 × 10^−2^
H0: Mean of energy is equal across all variants	10.1827	7.0220 × 10^−2^
H0: Standard deviation of energy is equal across all variants	3.9343	5.5891 × 10^−1^
**Tests applied to receptors**		
H0: Energy is equal across both receptors	0.0042	9.4828 × 10^−1^
H0: Mean of energy is equal across both receptors	0.0470	8.2845 × 10^−1^
H0: Standard deviation of energy is equal across both receptors	0.0404	8.4075 × 10^−1^
**Tests applied to combinations**		
H0: Energy is equal across all combinations	40.2081	3.2958 × 10^−5^
H0: Mean of energy is equal across all combinations	13.3356	2.7196 × 10^−1^
H0: Standard deviation of energy is equal across all combinations	8.3852	6.7844 × 10^−1^

The total number of samples is 120. The number of samples in each variant and in each receptor is 10. All statistical tests are performed at 5% significance level.

**Table 3:** Average energy by variant/receptor combination.

Variant/Receptor	Average Energy
Omicron/GRP78	−33.4403
Beta/GRP78	−33.0438
Delta/GRP78	−32.6514
Alpha/GRP78	−31.1821
Omicron/ACE2	−31.1089
Gamma/GRP78	−30.3382
Gamma/ACE2	−30.1941
Zeta/GRP78	−29.8385
Alpha/ACE2	−29.0857
Beta/ACE2	−28.2156
Zeta/ACE2	−27.9563
Delta/ACE2	−26.1153

## Data Availability

Data source is provided in references [18,19].

## Abbreviations

The following abbreviations are used in this manuscript:

ANOVA	Analysis of variance
COVID-19	Coronavirus disease 2019
DL	Deep learning
ML	Machine learning
*p*-value	Probability value
RBD	Receptor-binding domain
SARS-CoV-2	Severe acute respiratory syndrome coronavirus 2
